# Could brown bears (*Ursus arctos*) have survived in Ireland during the Last Glacial Maximum?

**DOI:** 10.1098/rsbl.2013.0281

**Published:** 2013-08-23

**Authors:** Saoirse A. Leonard, Claire L. Risley, Samuel T. Turvey

**Affiliations:** 1 Institute of Zoology, Zoological Society of London, Regent's Park, London NW1 4RY, UK; 2 University of Liverpool, Leahurst Campus, Neston, Cheshire CH64 7TE, UK

**Keywords:** hybridization, mammal extinction, migration, population viability analysis, refugium

## Abstract

Brown bears are recorded from Ireland during both the Late Pleistocene and early–mid Holocene. Although most of the Irish landmass was covered by an ice sheet during the Last Glacial Maximum (LGM), Irish brown bears are known to have hybridized with polar bears during the Late Pleistocene, and it is suggested that the Irish brown bear population did not become extinct but instead persisted *in situ* through the LGM in a southwestern ice-free refugium. We use historical population modelling to demonstrate that brown bears are highly unlikely to have survived through the LGM in Ireland under any combination of life-history parameters shown by living bear populations, but instead would have rapidly become extinct following advance of the British–Irish ice sheet, and probably recolonized Ireland during the end-Pleistocene Woodgrange Interstadial from a closely related nearby source population. The time available for brown bear–polar bear hybridization was therefore restricted to narrow periods at the beginning or end of the LGM. Brown bears would have been extremely vulnerable to extinction in Quaternary habitat refugia and required areas substantially larger than southwestern Ireland to survive adverse glacial conditions.

## 1. Introduction

The origin of Ireland's modern terrestrial vertebrate fauna is the subject of ongoing debate [1–3]. Late Quaternary Irish fossil deposits contain diverse mammal assemblages and reveal the former occurrence of several species, including the now regionally extinct brown bear (*Ursus arctos*), during both the Late Pleistocene and early–mid Holocene [4]. However, most of the Irish landmass was covered by an ice sheet for the majority of the Last Glacial Maximum (LGM) between 27 and 15 kya [5–7]. Many modern Irish animal groups are known to have become locally extirpated during this extreme climatic interval and recolonized during the subsequent Woodgrange Interstadial (end-Pleistocene) or early Holocene, probably via transient landbridges or anthropogenic introduction [3,4]. However, a small habitat refugium in southwestern Ireland probably remained ice-free throughout the LGM [5], suggesting that some cold-adapted terrestrial lineages may instead have persisted *in situ* across the Late Pleistocene–Holocene. This alternative hypothesis has received support from genetic analyses of some Irish small mammals [8] and amphibians [9].

Although no Irish brown bear fossils are known from the LGM (they are absent from the Irish fossil record between 26 340 ± 320 and 12 143 ± 46 year BP [10]), it has been proposed that bears may also have survived this interval in the Irish refugium at the northwestern periphery of their former European range [3] before finally becoming extinct in Ireland around 3000 year BP [10]. Ancient DNA analysis has recently shown that Pleistocene and Holocene Irish brown bear matrilines cluster together, suggestive of population continuity in Ireland across the Late Quaternary [10]. Interestingly, the inferred common matrilineal ancestor of modern polar bears (*Ursus maritimus*) also falls within the genetic diversity of Irish brown bears, indicating that this brown bear population hybridized with polar bears; it is suggested that hybridization may have occurred when the British–Irish ice sheet reached its maximum extent 22–20 kya and provided suitable polar bear habitat in Ireland [10].

Spatial patterns of species range change in response to natural or human-mediated environmental change remain incompletely understood. Broad taxon-focus and species-specific analytical studies of ‘dynamic biogeography’ have demonstrated that many species persist in peripheral subpopulations rather than core areas of their geographical range [11,12], although extinction rates may also be higher at range edges [13,14], making it difficult to predict the likelihood of brown bear survival in the peripheral Irish refugium. Understanding the dynamics of Late Pleistocene megafaunal extinctions also represents a long-standing problem in Quaternary research [15,16]. Geographical restriction to small environmental refugia during the Late Pleistocene is associated with greatly elevated risk of extinction in Europe's large mammal fauna [16,17], and population-level extinctions in response to Quaternary climatic fluctuations are increasingly being demonstrated in many European mammals [18]; however, relict populations of large-bodied mammals are known to have persisted for several millennia in some small, isolated island refugia [19]. To obtain new insights into Late Pleistocene mammalian population dynamics, and to clarify the Quaternary history of the Irish mammal fauna and the evolutionary history of bears in Europe, we therefore conducted population viability analysis (PVA) to determine whether *in situ* Irish brown bear survival was possible for the duration of the LGM.

## 2. Material and methods

**Figure 1.**
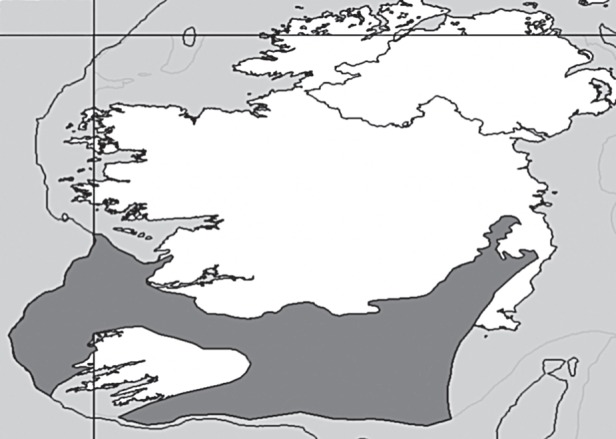
Maximum area of Irish refugium during the LGM (dark grey), incorporating the sea level drop reported in [5] and showing −90 m (light) and −100 m (dark) depth contours.

We conducted historical PVA using Vortex [20], a modelling program designed specifically for mammalian and avian populations with low fecundity and long life spans. This analytical approach has been used to investigate past population persistence for other now-extinct mammals [17,21]. Brown bear life-history traits (e.g. population density, reproductive rate) vary depending on environmental conditions, according to three main habitat types: Arctic barren-ground (latitudes more than 65° N) and continental interior (altitudes more than 1000 m), both with low primary productivity and high seasonality; and coastal, with high primary productivity and low seasonality [22]. We incorporated data from a range of modern brown bear populations (see the electronic supplementary material) and constructed two models: a general model incorporating mean/commonest values for brown bear life-history parameters across all habitats, and a model only incorporating life-history data from barren-ground populations, on the assumption that this modern-day habitat is closest to steppe–tundra conditions in Ireland during the LGM [4,6]. The area of the Irish refugium was determined as 29 315 km^2^ in ArcView v. 9.3 using Ehlers & Gibbard [6], based on a current exposed landmass of 14 750 km^2^, and with LGM sea levels 90 m lower along southeastern and western coasts and 100 m lower along the southwestern coast (figure 1; [5]). We assumed a single bear population in both models and that the LGM Irish refugium was homogenous regarding suitability for bear occupation, because brown bears are adaptable and capable of living on most terrains [22]. However, brown bear range size varies greatly with habitat productivity, with largest home ranges found on unproductive Arctic tundra [23]; we used barren-ground range size data for both models. Home ranges for bears inhabiting unproductive environments overlap extensively [23], so we used the median of population density estimates in different habitats (575 individuals) to estimate refugial carrying capacity (see the electronic supplementary material), and assumed initial population size was at carrying capacity. In both models, 1000 iterations were run for 10 000 years.

## 3. Results

Modelled extinction probability for brown bears in the Irish refugium was 1, with relatively rapid extinction occurring in both models: median persistence was 1204 years (95% CI, 851–1505 years) for the general model, and 149 years (95% CI, 109–205 years) for the barren-ground model. Sensitivity analysis showed that interbirth interval and female mortality rate have greatest impact on population persistence (see the electronic supplementary material, figure S2); there is little difference in these parameters between barren-ground and coastal brown bear populations, due to higher levels of intraspecific competition in more productive environments [22], suggesting that our models were not compromised by preferential use of barren-ground life-history data. From 50 Latin hypercube samples of parameter space in our sensitivity analysis, only one (see the electronic supplementary material, figure S3) returned an expected extinction time of more than 3000 years, and many more returned very low values (less than 100 years). We thus remain confident in the robustness of our conclusion that brown bears were extremely unlikely to survive the LGM in Ireland.

## 4. Discussion

PVA is an analytical tool providing a modelled outcome from available parameter values, in this case modern-day populations [24,25]. As such, modelling cannot unequivocally demonstrate that brown bears would have disappeared from Ireland during the LGM. Alternative possible values for poorly understood parameters, notably lethal equivalents (see the electronic supplementary material), may permit longer-term population survival, necessitating further investigation into inbreeding effects in bears. However, outputs from our two main models and accompanying sensitivity analyses strongly suggest that bears could not have persisted *in situ*, and are likely to have recolonized Ireland during the Woodgrange Interstadial from a closely related source population (probably on unglaciated southern Britain). Although the area of Irish ice cover varied between 27 and 15 kya, with a possible retreat 24–23 kya followed by re-advance [7], the similar population dynamics shown by both of our models demonstrate that Irish brown bears would probably have disappeared even if they had only been restricted to the southwestern refugium for part of the LGM. This suggests that the time window for hybridization between brown bears and polar bears in Ireland would have been relatively short and probably occurred either at the beginning of the LGM or after brown bear recolonization when the ice sheet retreated. However, it is possible that environmental conditions prevailing during the LGM may have driven brown bear behavioural accommodation and transient occupation of ice-shelf habitats, leading to greater range overlap with polar bears for the period before their extirpation.

Our findings reveal that brown bears would have been extremely vulnerable to extinction when their populations were periodically restricted to habitat refugia during Quaternary climatic fluctuations and would have required areas substantially larger than southwestern Ireland for long-term survival through adverse glacial environmental conditions. Conversely, although we demonstrate that remnant brown bear populations are very unlikely to persist in restricted geographical refugia through long periods of Quaternary climate change, they may still be able to survive for several hundred years or more in the absence of any population connectivity. Although brown bear populations in northern Eurasia and North America are still large and contiguous, the species has been extirpated from much of the southern portion of its former range, and most surviving populations in western and central Europe, central Asia and the United States are now small and fragmented due to historical-era anthropogenic pressures (persecution, habitat conversion; [26]), in most cases probably for the first time in their evolutionary history [27]. Many of these remnant populations are now seriously threatened with extinction, and their long-term survival is very unlikely in the absence of appropriate conservation management [26]. However, unlike the Irish brown bear population, there is still sufficient time to develop effective recovery strategies to prevent their disappearance.
